# Analysis of widely targeted metabolites of the euhalophyte *Suaeda salsa* under saline conditions provides new insights into salt tolerance and nutritional value in halophytic species

**DOI:** 10.1186/s12870-019-2006-5

**Published:** 2019-09-06

**Authors:** Qiang Li, Jie Song

**Affiliations:** Shandong Provincial Key Laboratory of Plant Stress, College of Life Science, Shandong Normal University, 88 Wenhua East Road, Jinan, 250014 People’s Republic of China

**Keywords:** Antioxidant activity, Halophyte, Salinity, *Suaeda salsa*, Widely targeted metabolites

## Abstract

**Background:**

*Suaeda salsa* L. (*S. salsa*) is an annual euhalophyte with high salt tolerance and high value as an oil crop, traditional Chinese medicine and vegetable. However, there are few comprehensive studies on the metabolomics of *S. salsa* under saline conditions.

**Results:**

Seedlings of *S. salsa* were cultured with 0, 200 and 500 mM NaCl for two days. Then, widely targeted metabolites were detected with ultra performance liquid chromatography and tandem mass spectrometry. A total of 639 metabolites were annotated. Among these, 253 metabolites were differential metabolites. Salt treatment increased the content of certain metabolites, such as nucleotide and its derivates, organic acids**,** the content of amino acids, lipids such as α-linolenic acid, and certain antioxidants such as quercetin. These substances may be correlated to osmotic tolerance, increased antioxidant activity, and medical and nutritional value in the species.

**Conclusion:**

This study comprehensively analyzed the metabolic response of *S. salsa* under salinity from the perspective of omics, and provides an important theoretical basis for understanding salt tolerance and evaluating nutritional value in the species.

**Electronic supplementary material:**

The online version of this article (10.1186/s12870-019-2006-5) contains supplementary material, which is available to authorized users.

## Background

Soil salinization has seriously affected agricultural production and food security. More than 800 million hectares of land worldwide are affected by soil salinization, which accounts for approximately 6% of the world’s total land area, and this would result in reduced food production [1]. Meanwhile, the increasing amount of arable land lost to urban sprawl is forcing agricultural production into marginal areas [2]. Halophytes can be used as food, medicine and feed, and for restoring salinization and land contaminated with heavy metals.

Metabolite differences are directly correlated to the phenotype of an organism [3, 4]. The total number of metabolites in plants is approximately 200,000 [5, 6]. Changes in the type and amount of metabolites can show how the organism adapts to environmental changes [7, 8]. Metabolomics is a method of qualitatively and quantitatively analyzing all metabolites in an organism [6, 9]. Plant metabolism was disturbed under abiotic stress, and plants need to regulate metabolic levels to maintain basic metabolism and reach new homeostasis [10]. Hence, metabolomics was the most direct tool for studying this process [11, 12]. During this process, the change in primary metabolism was most pronounced and also showed the general trend of plant response to abiotic stress. It involved the accumulation of compatible solutes such as sugars and sugar alcohols, amino acids, etc. to cope with osmotic stress [13]. However, changed in secondary metabolism were more specific for different species and stress conditions, such as modification and interaction of enzyme proteins [14], increase in asparagine [15], accumulation of flavonoids and other phenols [16], to scavenge ROS and act as a signal regulator substance [13]. At present, metabolomics has been playing an important role in the analysis of regional differences in wild rice [17], plant tolerance to abiotic stress [10], nitrogen metabolism [18], and the phenotypic variation analysis of *Saccharomyces cerevisiae* [19]. For example, under salt stress, co-induced glycolysis and sucrose metabolism and the co-reduction of the methylation cycle occur in *Arabidopsis* under salt stress [20]. Furthermore, small molecular organic solutes involved in the resistance to osmotic stress in *Zea mays* are significantly induced at high salinity, and this is found to be stronger in the shoots, when compared to the roots [21]. In salt-tolerant *Hordeum vulgare* L., the levels of hexose phosphate, TCA circulating intermediates, and metabolites involved in cell protection increases with the increase in salt concentration [22]. These studies have found pathways and metabolites that play important roles in salt tolerance. Therefore, metabolomics provides an important basis for salt response studies in plants. *Suaeda salsa* (*S. salsa*) has stronger salt tolerance, and its metabolic level changes under salt stress. Hence, it has high value for research.

*S. salsa* is an euhalophyte with high salt tolerance during germination [23–26], vegetative growth [27] and reproductive stages [28–31]. The species is considered to have potential as a vegetable and oilseed crop [32, 33], and it is rich in protein, crude fiber, carotenoids and amino acids [34]. Furthermore, the species has high value as a medicine, and is a promising model for understanding salt tolerance [34]. The content of flavanols in *S. salsa* is much higher in July (98.8 mg g^− 1^ DW) than in the other months, and the extracts in July have the highest antioxidant activity in vitro [34]. *S. salsa* has high ability to maintain ion homeostasis. For example, *SsNHX1* and *SsSOS1* are involved in maintaining Na^+^ homeostasis, while *SsHKT1;1* is involved in maintaining K^+^ and *SsCAX1* is involved in maintaining Ca^2+^ homeostasis under salinity in *S. salsa* [34]. Salinity upregulated expression levels in certain genes, such as choline monooxygenase (CMO), betaine aldehyde dehydrogenase (BADH) and catalase (CAT), elevate the activities of superoxide dismutase (SOD), peroxidase (POD), CAT and glutathione peroxidase (GPx) in *S. salsa* [35]. High salinity causes metabolic responses, such as depleted amino acids, malate, fumarate, choline and phosphocholine, and elevated betaine and allantoin in the shoots, as well as depleted glucose and fructose, and elevated proline, citrate and sucrose in the roots of *S. salsa* [35]. A series of metabolomics studies have also been conducted in *S. salsa* under heavy metal stress at ambient salt concentrations [36–38]. However, there are few comprehensive studies on the metabolomics of *S. salsa* in controlled saline conditions, such as determining how salinity affects the metabolites in the species. Widely targeted metabolomics based on multiple reaction monitoring (MRM), using multiple ion monitoring (MIM) survey scans to trigger enhanced product ion (EPI) acquisition to identify metabolites. Compared to non-targeted metabolomics, it is a more sensitive and accurate method for detecting metabolites [39]. Therefore, widely targeted metabolites of *S. salsa* under salt stress were investigated in the present study.

## Results

### Data quality assessment

During the analysis, quality control (QC) samples prepared by mixing the sample extracts were inserted in every 10 test samples to monitor the reproducibility of the analysis process. The accuracy and reproducibility of metabolite detection could be determined using the superimposed display analysis of mass spectrometry total ion current (TIC). TIC is the spectrum obtained by continuously summing the intensity of all ions in the mass spectrum at each time point. The multi-substance extracted ion chromatogram (XIC) can be used determine the ion flux spectrum of each extracted substance in the multiple reaction monitoring mode (MRM). The mass spectral peaks for each color represents the different metabolites detected. The peak area represents the relative content of the corresponding substance. The integration and calibration of peaks were performed using the MultiaQuant software (v 3.0.3).

### Qualitative and quantitative metabolites

The qualitative and quantitative mass spectrometry analysis of metabolites in samples was performed on metabolites based on the Kyoto Encyclopedia of Genes and Genomes (KEGG) database, MetWare database (MWDB), and multiple reaction monitoring (MRM). A total of 639 metabolites were detected based on the metabolic analysis of widely targeted metabolites technique, which included 29 amino acids, 60 amino acid derivatives, 15 benzoic acid derivatives, three pyridine derivatives, nine alcohols and polyols, five cholines, eight catechin derivatives, 16 phenolamides, 53 nucleotide and its derivatives, 15 anthocyanins, 52 flavone, 32 flavonol, one flavonolignan, 29 flavone C-glycosides, 18 flavanone, nine isoflavones, 17 quinates and its derivatives, 32 hydroxycinnamoyl derivatives, six tryptamine derivatives, four alkaloids, 19 carbohydrates, two terpenoids, 16 vitamins, 17 coumarins, five nicotinic acid derivatives, eight indole derivatives, 63 organic acids, 63 lipids, and 33 other metabolites (Additional file 1: Table S1).

### Principal component analysis (PCA)

**Fig. 1:**
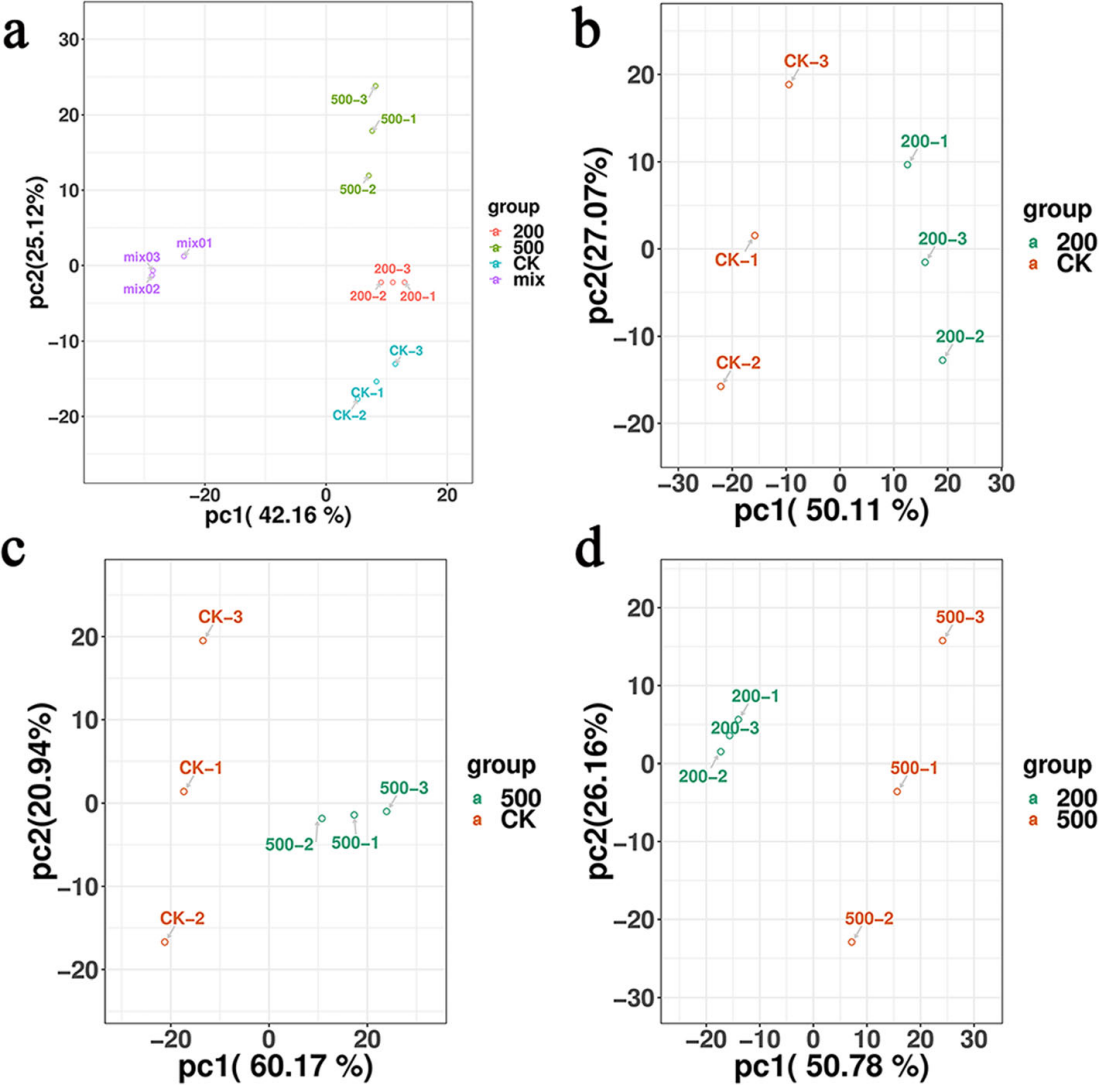
Principal component analysis (PCA) score map: (**a**) overall score scatter plot of the PCA model with QC, (**b**) PCA score map of the 200 mM NaCl vs. CK (0 mM NaCl), (**c**) 500 mM NaCl vs. CK, and (**d**) 500 mM NaCl vs. 200 mM NaCl

The PCA of the quality control and treatments revealed that the variability of each treatment of samples was small. The samples had similar metabolic characteristics, and the test results were stable and reproducible. In addition, the separation trend between treatments was obvious, indicating the significant metabolic differences between salt treatments (Fig. 1a). The metabolism of these three treatments was clearly separated in the first component (PC1), and the effect of salt treatment on the metabolism of *S. salsa* was obvious (Fig. 1b, c and d).

### Orthogonal partial least squares-discriminant analysis (OPLS-DA)

**Fig. 2:**
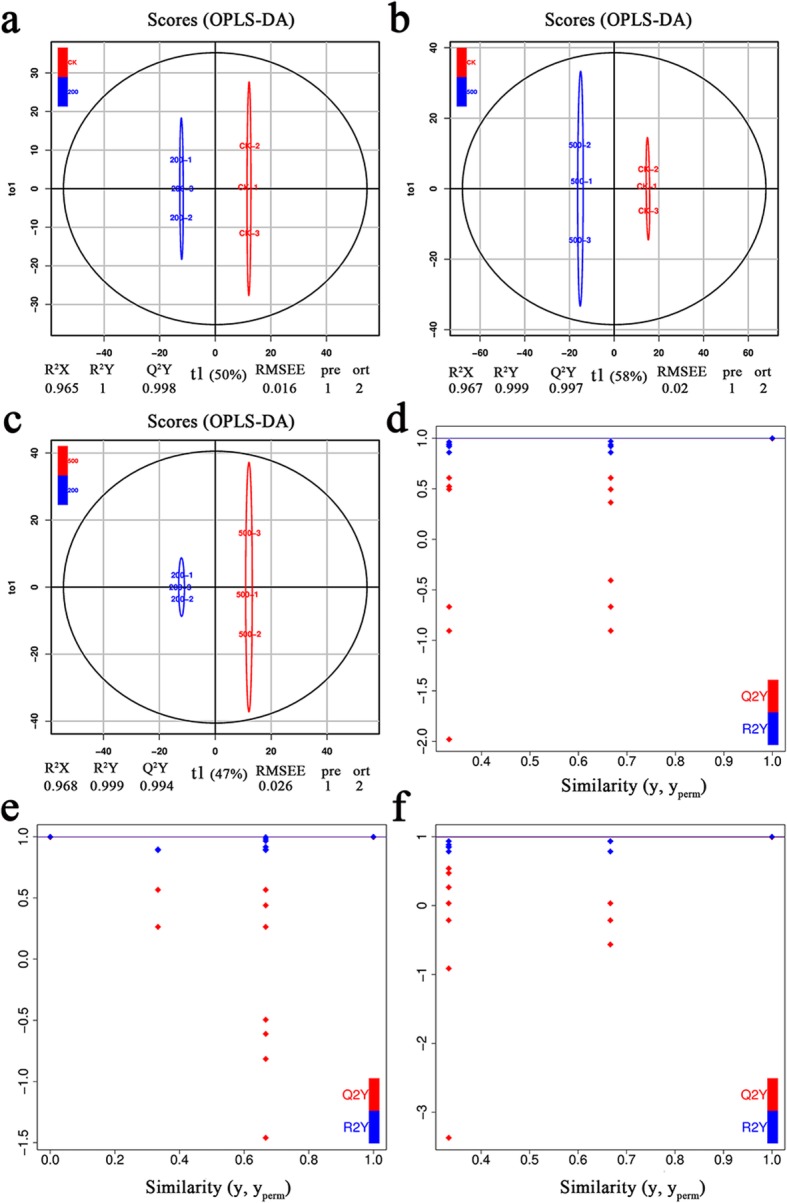
OPLS-DA scores and permutation verification: scores of the OPLS-DA model with (**a**) 200 mM NaCl vs. CK (0 mM NaCl), (**b**) 500 mM NaCl vs. CK, and (**c**) 500 mM NaCl vs. 200 mM NaCl; OPLS-DA permutation analysis model verification chart of (**d**) 200 mM NaCl vs. CK, (**e**) 500 mM NaCl vs. CK, and (**f**) 500 mM NaCl vs. 200 mM NaCl. R^2^Y and Q^2^ represent the interpretation rate of the model to the Y matrix and the prediction ability of the model, respectively. A value closer to 1 means that the model is more stable and reliable, and when Q^2^ is > 0.9, the model is excellent. The horizontal line corresponds to the R^2^ and Q^2^ of the original model, and the red and blue points represent the R^2^’ and Q^2^’ of the model after Y replacement, respectively

The OPLS-DA decomposes the X matrix information into Y correlation and irrelevance by orthogonal signal correction (OSC) and partial least squares discriminant analysis (PLS-DA) [40]. The difference variables were filtered by eliminating the irrelevant differences. Compared with PCA, PLS-DA can maximize the distinction between treatments, and is more conducive to finding differential metabolites. The R^2^Y and Q^2^ scores were all greater than 0.99 in the 200 mM NaCl vs. CK, 500 mM NaCl vs. CK, and 500 mM NaCl vs. 200 mM NaCl (Fig. 2a, b and c), demonstrating that the results of the salt treatment that led to the differential metabolism of *S. salsa* were correct.

The OPLS-DA model was verified using 200 alignment experiments. The horizontal line corresponded to the R^2^ and Q^2^ of the original model, while the red and blue dots represented the R^2^’ and Q^2^’ after replacement, respectively. These results show that the model was meaningful, and that the differential metabolites could be screened according to the VIP value analysis in the subsequent analysis (Fig. 2d, e and f).

### Hierarchical cluster analysis (HCA) and volcano plot

**Fig. 3:**
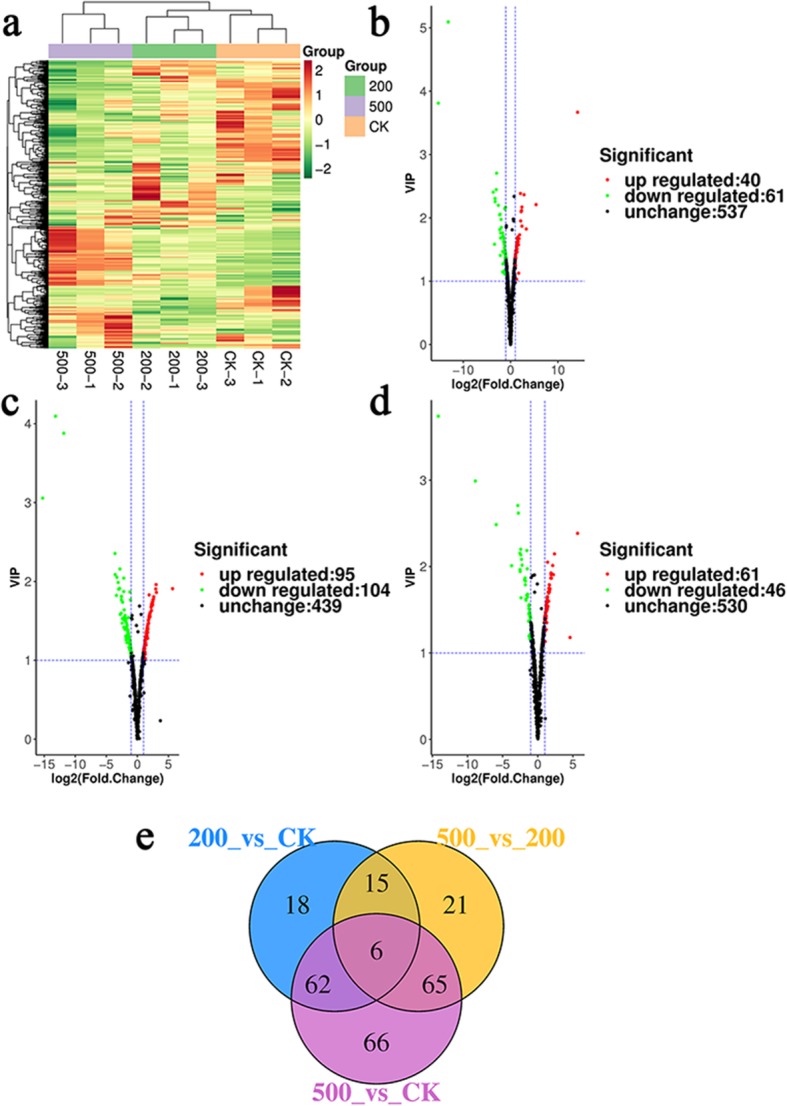
Hierarchical cluster analysis (HCA), and volcano plot and venn diagram: (**a**) the heat map shows the differential expression of metabolites between CK (0 mM NaCl), 200 and 500 mM NaCl. The green color indicates the decrease of differentially expressed metabolites, while the red color indicates the increase of differentially expressed metabolites; the volcano plot: (**b**) 200 mM NaCl vs. CK, (**c**) 500 mM NaCl vs. CK, and (**d**) 500 mM NaCl vs. 200 mM NaCl. The green dots in the figure represent the differentially expressed metabolites that were decreased, the red dots represent the increased differentially expressed metabolites, and the black color indicates the detected metabolites, but there was no significant difference; (**e**) the Venn diagram shows overlapping and specific differential metabolites from CK, 200 and 500 mM NaCl

The HCA can evaluate differences in the characteristics of salt treatment that lead to metabolite accumulation, and comprises of intra-treatment homogeneity and inter-treatment variability. With the increase in NaCl concentration, the difference in the expression of metabolites increased (Fig. 3a). The points in the volcano map represents the metabolites, and the abscissa and ordinate represents the logarithm and VIP values of the quantitative difference of metabolites in the two samples, respectively. Metabolites with a fold change of ≥2, a fold change of ≤0.5, and a VIP of ≥1 were selected. The metabolites screened under the above conditions had significant differences. There was no difference in the expression of most metabolites, and the number of increases in differentially expressed metabolites was close to the number of decreases (Fig. 3b, c and d).

### Statistical analysis of differential metabolites

There were 253 metabolites with different content in all 639 metabolites (Additional file 1: Table S1). These were divided into 10 categories: amino acid and its derivatives, phenolamides and phenolic acids, nucleotide and its derivates, flavonoids, lipids, carbohydrates, vitamins, indole derivatives, organic acids, and others (Table 1). Compared to controls, there were 101 differential metabolites in the 200 mM NaCl treatment, in which 61 metabolites were decreased, while 40 metabolites were increased. Furthermore, there were 199 differential metabolites in the 500 mM NaCl treatment, in which 104 metabolites were decreased, while 95 metabolites were increased. In addition, there were 107 differential metabolites in the 500 mM NaCl treatment, when compared to the 200 mM NaCl treatment, in which 46 metabolites were decreased, while 61 metabolites were increased (Table 1 and Additional file 2: Table S2).

**Table 1:** Numbers of differential metabolites in the leaves of *S. salsa*. Plants were cultured at 0, 200 and 500 mM NaCl for two days

Group name Class	200 vs 0		500 vs 200		500 vs 0	
	Up	Down	Up	Down	Up	Down
Amino acid and its derivatives	1	13	14	3	13	14
Phenolamides and phenolic acids	6	8	4	7	5	13
Nucleotide and its derivates	5	2	11	8	18	11
flavonoids	9	18	5	14	14	31
Lipids	4	2	19	2	25	3
Others	3	6	4	6	6	13
Carbohydrates	2	1		1	2	3
Vitamins	1	2		2	2	6
Indole derivatives	1	1	3	1	1	2
Organic acids	8	8	1	2	9	8
Sig diff	40	61	61	46	95	104
All sig diff	101		107		199	

A total of 68 metabolites differed between the low-salt and high-salt treatment, when compared to controls, in which 45 metabolites were decreased, while 22 metabolites were increased. However, 4-(aminomethyl)-5-(hydroxymethyl)-2-methylpyridin-3-ol and dihydromyricetin were barely detected in the 200 mM and 500 mM NaCl treatments. The most decreased substances were tangeretin, L-ascorbate, 3-hydroxy-3-methylpentane-1, and 5-dioic acid (LogFC < − 2.5), while the most increased substance was 2′-hydroxygenistein (LogFC > 2.8). Moreover, imidazole-4-acetate was increased after the 200 mM NaCl treatment, but was decreased after the 500 mM NaCl treatment (Additional file 3: Table S3).

### Functional annotation and enrichment analysis of differential metabolites

**Fig. 4:**
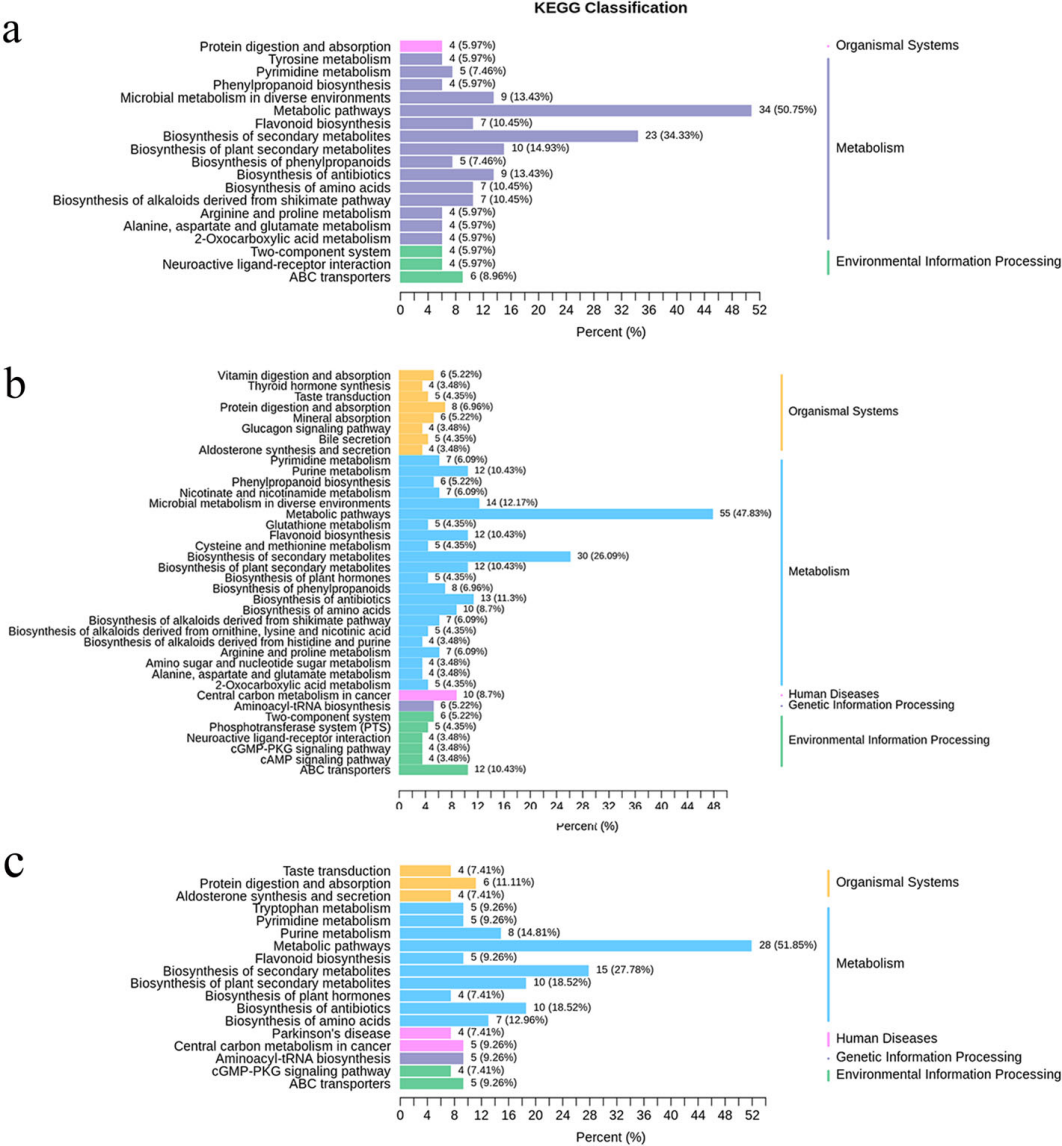
Functional annotation and enrichment analysis of certain important differential metabolites: the ordinate was the name of the KEGG metabolic pathway, and the abscissa was the number of metabolites annotated to the pathway and its proportion to the total number of annotated metabolites in the function annotation: (**a**) 200 mM NaCl vs. CK (0 mM NaCl), (**b**) 500 mM NaCl vs. CK, and (**c**) 500 mM NaCl vs. 200 mM NaCl

**Fig. 5:**
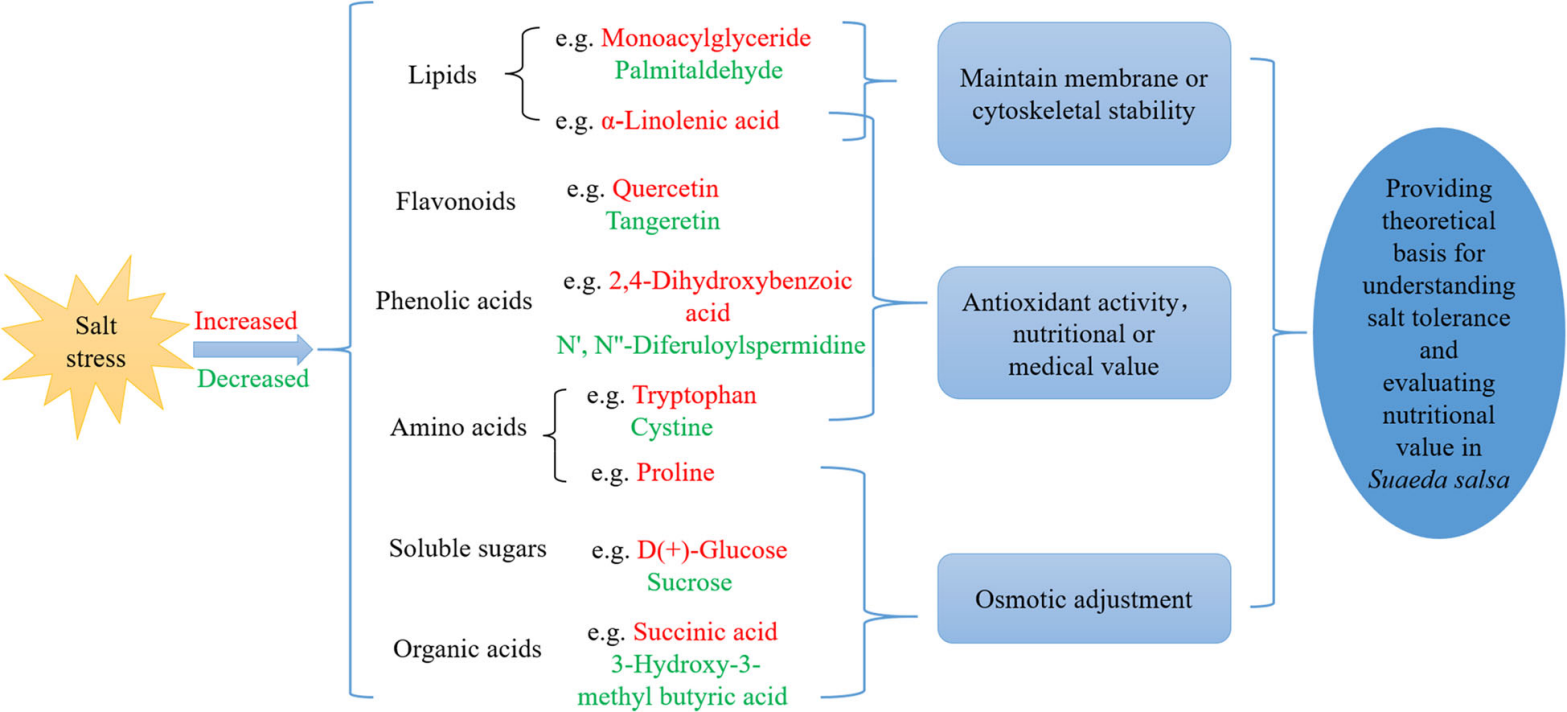
The outline for metabolic response of *S. salsa* under salinity stress. The red represents increased, while the green represents decreased

The results of these differentially significant metabolite annotations were classified according to the type of pathway in the KEGG database (http://www.genome.jp/kegg/). Differential metabolites were mainly involved in the metabolic pathways and biosynthesis of secondary metabolites, such as flavonoids, phenols and phenolic acids, amino acids and their derivatives, lipids, organic acids, and other small molecules (Fig. 4a, b and c, Additional file 4: Table S4). The changes in content of these metabolites may play an important role in cell membrane structure protection, maintaining cell osmotic potential and resisting the destruction of reactive oxygen species (ROS) (Fig. 5).

## Discussion

Seeds of *S. salsa* contain high oil, and are rich in unsaturated fatty acids, especially linoleic acid. Hence, this can be used as a source of high-quality edible oil [32, 33]. Fatty acid methyl esters extracted from seedlings and seeds, especially 9, 12-octadecandienoic acid methyl ester and γ-linolenic acid methyl ester, can inhibit the formation of inflammatory factors, and have obvious anti-inflammatory effects [34]. Furthermore, *S. salsa* can enrich the heavy metals in the soil, such as Cu, Zn, Pb and As, in order to restore contaminated land, and has high value as the modal for understanding plant salt tolerance [34]. However, the comprehensive metabolic response of *S. salsa* under saline conditions has not been investigated.

Euhalophytes decrease their water potential by accumulating organic substances with small molecules and inorganic ions to resist external osmotic stress [34, 41–44]. Soluble sugars and organic acids play important roles in decreasing water potential for plants under osmotic stress [45]. In the present study, salinity induced the increase of certain kinds of soluble sugars and organic acids, when compared to controls (Table 1). Under osmotic stress, the study conducted by Cao et al. (2004) revealed a significant increase in the content of certain free amino acids, including proline (Pro), aspartic acid (Asp), phenylalanine (Phe) and alanine (Ala), in maize [46]. In the present study, high salinity (500 mM) induced the increase of amino acids, when compared to low salinity (200 mM) (Table 1). This means that *S. salsa* under high salinity can accumulate more amino acids, as well as soluble sugars and organic acids, which can decrease the osmotic potential, and help the species deal with osmotic stress. Furthermore, these amino acids can also increase the nutritional value in species such as vegetables. It has been shown that 3-methylcrotonyl glycine is an acyl glycine that has been studied in animals, but this is not been reported in plants [47]. Interestingly, 3-methylcrotonyl glycine was detected in *S. salsa* at 200 mM NaCl, but was barely detected in controls and at 500 mM NaCl (Additional file 1: Table S1). Hence, the physiological role and molecular mechanism for the metabolism of the substance in *S. salsa* can be further investigated.

Lipids mediate some important mechanisms to deal with salt stress, such as the process of participating in cell metabolism and maintaining the stability of the cytoskeleton [48–51]. In the present study, 500 mM NaCl induced the significant differential expression of lipid metabolites, and more unsaturated fatty acids were increased, when compared to those in controls and at 200 mM NaCl (Table 1). Monoacylglyceride (MAG), lysophosphatidylcholine (LysoPC), octadecatrienoic acid’s derivates, α-linolenic acid (ALA) and punicic acid are unsaturated fatty acids that are good for human health, especially ALA [52]. The main role of ALA may be as a precursor to long-chain n-3 PUFAs, such as EPA and DHA [53]. ALA may have a function of preventing heart disease and sudden cardiac death [54, 55]. The increase of α-linolenic acid content plays an important role in regulating intracellular fatty acid unsaturation to resist salt stress [56]. In the present study, 500 mM NaCl increased the content of α-linolenic acid, and the products related to metabolic pathways also significantly increased (Additional file 2: Table S2). Hence, the increased content of ALA may play a role in salt tolerance. Meanwhile, this means that planting *S. salsa* under high salinity should increase its nutritional value as edible vegetables.

**Fig. 6:**
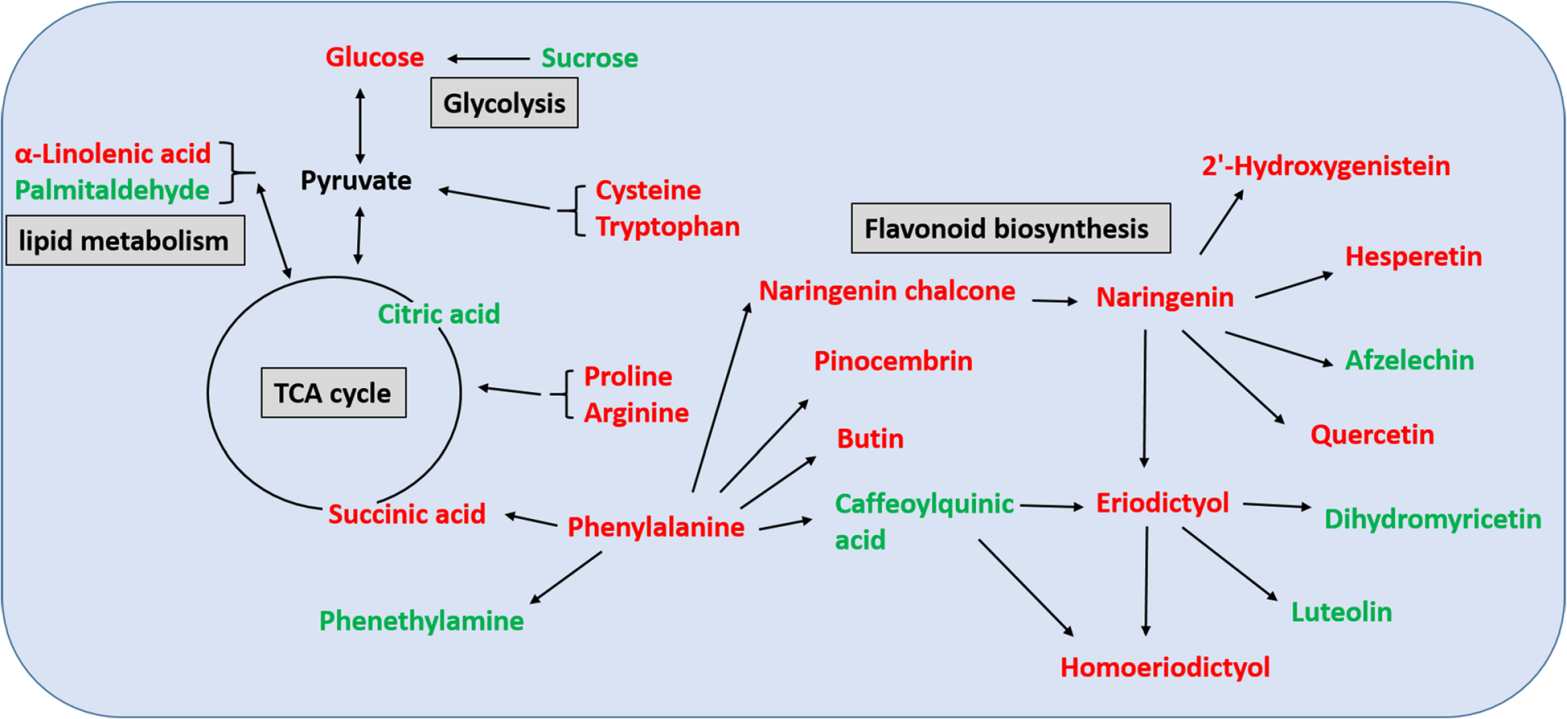
Schematic presentation of the pathway for certain important metabolites as affected by salinity in *S. salsa.* The red represents increased, while the green represents decreased

Polyphenols are generally divided into two categories: flavonoids (e.g. flavones, flavanones, flavonols and catechins) and phenolic acids (e.g. hydroxybenzoic acid, hydroxycinnamic acid and quinic acid) [57–59]. Polyphenols have high antioxidant activity, and can prevent cardiovascular diseases and cancers [60, 61]. Therefore, these have been applied in functional foods, cosmetics and medicine [62]. In the present study, different contents of 60 flavonoids and 28 phenolic acids under salt treatment were detected (Additional file 2: Table S2). The content of 5-O-p-coumaroylquinic acid, N-acetyl tryptamine, 2′-hydroxygenistein, morin, 2,4-dihydroxybenzoic acid, N′, N″, N″‘-p-coumaroyl-cinnamoyl-caffeoyl spermidine, butin, quercetin, isorhamnetin, homoeriodictyol, hesperetin, naringenin, protocatechuic aldehyde and phloretin significantly increased with salinity. Among these, certain substances have special functions. For example, quercetin is an anti-oxidative flavonoid widely distributed in plants, and is a promising agent for cancer prevention [63–66]. However, more antioxidants, such as flavonoids and polyphenols, were decreased by salinity in the present study (Table 1). In *S. salsa*, the activities of Mn-SOD and several isoforms of Fe-SOD and CuZn-SOD increased under salinity [67]. The overexpression of *Ss.sAPX* (a gene of the stromal APX in *S. salsa*) increased the salt tolerance of transgenic *Arabidopsis* plants during both the germination and vegetative growth stages [68]. *S. salsa* is highly salt tolerant, and grows as well with 400 mM NaCl, when compared to 10 mM NaCl [69]. The distinctive trait of salt tolerance in euhalophytes, such as *S. salsa*, is to maintain ion homeostasis, including Na^+^ accumulation in the vacuoles [70], Na^+^ and Cl^−^ exclusion through the roots [70, 71], and K^+^ [72] and Ca^2+^ [73] homeostasis. Scavenging ROS may be the subsequent mechanism in salt tolerance only when ion homeostasis is destroyed in *S. salsa*. In addition, *S. salsa* may scavenge ROS, and mainly relies on enzymatic components, rather than on non-enzymatic components. The role of enzymatic and non-enzymatic components in scavenging ROS should be further investigated. Based on the present results, this study demonstrated that the reconstitution of the metabolic homeostasis of *S. salsa* under salt stress included: 1) the accumulation of primary metabolites (e.g. amino acids, soluble sugars, organic acids, lipids) exerts resistance to osmotic stress and maintains the osmotic potential and metabolism of cells; 2) secondary metabolites may play an important role as antioxidants and regulatory substances including quercetin, 2,4-dihydroxybenzoic acid, isorhamnetin, 2′-hydroxygenistein, and other metabolites that were significantly inhibited or induced by salt. These metabolites have special nutritional and medicinal value, and also greatly increase the application value of *S. salsa* (Fig. 6).

## Conclusion

The present study used widely targeted metabolites based on the UPLC-MS/MS detection platform to analyze the metabolic differences of *S. salsa* under different NaCl concentrations. The present study comprehensively analyzed the metabolic response of *S. salsa* under salt stress from the perspective of omics, providing an important theoretical basis for understanding salt tolerance and evaluating the nutritional value of *S. salsa*.

## Methods

### Samples

The seeds of *S. salsa* were obtained from Dongying Research Academy of Agriculture Science, China. Dry seeds were stored in a fridge at < 4 °C before use.

In late March 2018, the brown seeds of *S. salsa* were planted in each plastic pot, which had 2 kg of rinsed river sand. The seedlings were cultured in a glasshouse under natural light. The temperature was 24 ± 4 °C during the day and 18 ± 4 °C at night in the glasshouse. The seedlings were watered every day with 1 mM of NO_3_^−^-N nutrient solution [70]. The pH of the solution was adjusted to 6.2 ± 0.1 with KOH and H_2_SO_4_.

After the seedlings were pre-cultured for 50 days, 15 seedlings in each pot were left and treated with 0 (as a control, which is indicated as CK in the figures), 200 and 500 mM NaCl (three pots for each concentration of NaCl), which was prepared with 1 mM NO_3_^−^-N nutrient solution [70]. In order to avoid osmotic shock, NaCl was added in increments of 50 mM per day. After the highest salinity concentration was obtained for two days, the matured fresh leaves in the same position were harvested and frozen in liquid nitrogen. Then, the samples were stored in dry ice and mailed to MetWare for metabolite testing. For each concentration of NaCl, the matured fresh leaves from each pot were harvested for metabolite testing. That is, three replicates were set for each NaCl treatment.

### Sample extraction process

The leaves were first freeze-dried and then grounded to powder using a grinder (MM 400; Retsch, Germany) at 30 Hz for 1.5 min. Then, 0.1 g of the powder was placed in 1 ml of 70% aqueous methanol at 4 °C overnight, and vortexed for three times during the period to increase the extraction efficiency. Next, the extract was centrifuged at 10,000 g for 10 min, and the supernatant was filtered through a microporous membrane (0.22-μm pore size) before LC–MS/MS analysis.

### Analysis of metabolites by ultra performance liquid chromatography and tandem mass spectrometry

The ultra-performance liquid chromatography (UPLC) (Shim-pack UFLC SHIMADZU CBM30A, http://www.shimadzu.com.cn/) and tandem mass spectrometry (MS/MS) (Applied Biosystems 6500 QTRAP) conditions were as follows: column, waters ACQUITY UPLC HSS T3 C18 1.8 μm, 2.1 × 100 mm; mobile phase, the aqueous phase was ultrapure water (0.04% acetic acid), while the organic phase was acetonitrile (0.04% acetic acid); gradient of water/acetonitrile, 95:5 V/V for zero minutes, 5:95 V/V for 11.0 min, 5:95 V/V for 12.0 min, 95:5 V/V for 12.1 min, and 95:5 V/V for 15.0 min; flow rate of 0.4 ml/min; column temperature at 40 °C; injection volume at 2 μl. The electrospray ionization (ESI) temperature was 500 °C, the mass spectrometry voltage was 5500 V, the curtain gas (CUR) was 25 psi, and the collision induced dissociation (CAD) parameter was set as high. In the triple quadrupole (QQQ), each ion pair was scanned for detection based on the optimized decompression potential (DP) and collision energy (CE) [39].

### Qualitative and quantitative determination of metabolites

Based on the public metabolite database (e.g. MassBank or KNApSAcK) and the self-built database MetWare database (MWDB), the material was qualitative by secondary spectral information, while the isotope signal and the repetitive signal are removed during the analysis.

The metabolites were quantified using multiple reaction monitoring (MRM) of triple quadrupole mass spectrometry. The ions corresponding to other molecular weight substances were excluded, and the precursor ions of the target substance were screened. Meanwhile, in the collision cell, the precursor ions were ionized to break and form fragment ions, and the characteristic fragment ions were selected by triple quadrupole filtration. This makes the quantitative results more accurate and repeatable. Peak area integration was performed on the obtained metabolite mass spectral peaks, and the mass spectral peaks of the metabolites in different samples were integrated [74].

### Statistical analysis

Multivariate statistical analysis methods, including principal component analysis (PCA) and orthogonal partial least squares discriminant analysis (OPLS-DA), were used. PCA was used to recombine original variables into new, mutually independent variables through orthogonal transformations, revealing the internal structure of multiple variables through a few principal components [75]. Based on the results of the OPLS-DA, a multivariate analysis of variable importance in the project (VIP) in the OPLS-DA model could be used to initially screen for metabolites with differences. At the same time, differential metabolites could be further screened by combining the *P*-values or fold changes in the univariate analysis. Finally, the differential metabolites were precisely mined. Hierarchical cluster analysis (HCA) was performed on the accumulation patterns of metabolites between different samples using the R software (www.r-project.org/). The KEGG database was used to annotate the differential metabolites [76].

## Additional files

Additional file 1: **Table S1.** All detected metabolites in the leaves of *S. salsa* treated with different concentrations of NaCl. (XLSX 109 kb)

Additional file 2: **Table S2.** All differential metabolites in the leaves of *S. salsa* treated with different concentrations of NaCl. (XLSX 68 kb)

Additional file 3: **Table S3.** Differential metabolites in the leaves of *S. salsa* at salinity, when compared to controls. (XLSX 30 kb)

Additional file 4: **Table S4.** Differentially significant metabolite annotations classified by KEGG pathways. (XLSX 44 kb)

## Data Availability

The datasets used and/or analysed during the current study available from the corresponding author on reasonable request.

## Abbreviations

MRM: Multiple reaction monitoring
MS/MS: Tandem mass spectrometry
QQQ: Triple quadrupole
UPLC: Ultra performance liquid chromatography
VIP: Variable importance in the project

## References

[1] Munns R (2005). Genes and salt tolerance: bringing them together. New Phytol.

[2] Shabala S (2013). Learning from halophytes: physiological basis and strategies to improve abiotic stress tolerance in crops. Ann Bot.

[3] Fiehn O, Kopka J, Dörmann P, Altmann T, Trethewey RN, Willmitzer L (2000). Metabolite profiling for plant functional genomics. Nat Biotechnol.

[4] Fukusaki E, Kobayashi A (2005). Plant metabolomics: potential for practical operation. J Biosci Bioeng.

[5] Fiehn O, Kloska S, Altmann T (2001). Integrated studies on plant biology using multiparallel techniques. Curr Opin Biotech.

[6] Weckwerth W (2003). Metabolomics in systems biology. Annu Rev Plant Biol.

[7] Fiehn O (2002). Metabolomics-the link between genotypes and phenotypes. Plant Mol Biol.

[8] Töpfer N, Kleessen S, Nikoloski Z (2015). Integration of metabolomics data into metabolic networks. Front Plant Sci.

[9] Dettmer K, Aronov PA, Hammock BD (2007). Mass spectrometry-based metabolomics. Mass Spectrom Rev.

[10] Arbona V, Manzi M, Ollas CD, Gómez-Cadenas A (2013). Metabolomics as a tool to investigate abiotic stress tolerance in plants. Int J Mol Sci.

[11] Shulaev V, Cortes D, Miller G, Mittler R (2008). Metabolomics for plant stress response. Physiol Plantarum..

[12] Hong J, Yang L, Zhang D, Shi J (2016). Plant metabolomics: an indispensable system biology tool for plant science. Int J Mol Sci.

[13] Obata T, Fernie AR (2012). The use of metabolomics to dissect plant responses to abiotic stresses. Cell Mol Life Sci.

[14] Durek P, Walther D (2008). The integrated analysis of metabolic and protein interaction networks reveals novel molecular organizing principles. BMC Syst Biol.

[15] Sanchez DH, Pieckenstain FL, Szymanski J, Erban A, Bromke M, Hannah MA, Kraemer U, Kopka J, Udvardi MK (2011). Comparative functional genomics of salt stress in related model and cultivated plants identifies and overcomes limitations to translational genomics. PLoS One.

[16] D’Auria JC, Gershenzon J (2005). The secondary metabolism of *Arabidopsis thaliana*: growing like a weed. Curr Opin Plant Biol.

[17] Yan N, Du Y, Liu X, Chu MJ, Shi J, Zhang HB, Liu YH, Zhang ZF (2019). A comparative UHPLC-QqQ-MS-based metabolomics approach for evaluating Chinese and north American wild rice. Food Chem.

[18] Kusano M, Fukushima A, Redestig H, Saito K (2011). Metabolomic approaches toward understanding nitrogen metabolism in plants. J Exp Bot.

[19] Kato H, Izumi Y, Hasunuma T, Matsuda F, Kondo A (2012). Widely targeted metabolic profiling analysis of yeast central metabolites. J Biosci Bioeng.

[20] Kim JK, Bamba T, Harada K, Fukusaki E, Kobayashi A (2007). Time-course metabolic profiling in *Arabidopsis thaliana* cell cultures after salt stress treatment. J Exp Bot.

[21] Gavaghan CL, Li JV, Hadfield ST, Hole S, Nicholson JK, Wilson ID, Howe PWA, Stanley PD, Holmes E (2011). Application of NMR-based metabolomics to the investigation of salt stress in maize (*Zea mays*). Phytochem Analysis.

[22] Widodo PJH, Newbigin ED (2009). Tester M, Bacic a, Roessner U. metabolic responses to salt stress of barley (*Hordeum vulgare* L.) cultivars, Sahara and clipper, which differ in salinity tolerance. J Exp Bot.

[23] Li WQ, Yamaguchi S, Khan MA, An P, Liu XJ, Tran LSP (2016). Roles of gibberellins and abscisic acid in regulating germination of *Suaeda salsa* dimorphic seeds under salt stress. Front Plant Sci.

[24] Song J, Shi WW, Liu RR, Xu YG, Sui N, Zhou JC, Feng G (2017). The role of the seed coat in adaptation of dimorphic seeds of the euhalophyte *Suaeda salsa* to salinity. Plant Spec Biol..

[25] Xu YG, Liu RR, Sui N, Shi WW, Wang L, Tian CY, Song J (2016). Changes in endogenous hormones and seed coat phenolics during seed storage of two *Suaeda salsa* populations. Aust J Bot.

[26] Zhou JC, Fu TT, Sui N, Guo JR, Feng G, Fan JL, Song J (2016). The role of salinity in seed maturation of the euhalophyte *Suaeda salsa*. Plant Biosyst..

[27] Chen TS, Yuan F, Song J, Wang BS (2016). Nitric oxide participates in waterlogging tolerance through enhanced adventitious root formation in the euhalophyte *Suaeda salsa*. Funct Plant Biol.

[28] Guo JR, Suo SS, Wang BS (2015). Sodium chloride improves seed vigour of the euhalophyte *Suaeda salsa*. Seed Sci Res.

[29] Guo JR, Li YD, Han GL, Song J, Wang BS (2018). NaCl markedly improved the reproductive capacity of the euhalophyte *Suaeda salsa*. Funct Plant Biol.

[30] Wang FX, Xu YG, Wang S, Shi WW, Liu RR, Feng G, Song J (2015). Salinity affects production and salt tolerance of dimorphic seeds of *Suaeda salsa*. Plant Physiol Bioch..

[31] Wang FX, Yin CH, Song YP, Li Q, Tian CY, Song J (2018). Reproductive allocation and fruit-set pattern in the euhalophyte *Suaeda salsa* in controlled and field conditions. Plant Biosyst..

[32] Zhao YQ, Ma YC, Duan HM, Liu RR, Song J (2019). Traits of fatty acid accumulation in dimorphic seeds of the euhalophyte *Suaeda salsa* in saline conditions. Plant Biosyst..

[33] Zhao YQ, Ma YC, Li Q, Yang Y, Guo JR, Song J (2018). Utilization of stored lipids during germination in dimorphic seeds of euhalophyte *Suaeda salsa*. Funct Plant Biol.

[34] Song J, Wang BS (2015). Using euhalophytes to understand salt tolerance and to develop saline agriculture: *Suaeda salsa* as a promising model. Ann Bot.

[35] Wu HF, Liu XL, You LP, Zhang LB, Zhou D, Feng JH, Zhao JM, Yu JB (2012). Effects of salinity on metabolic profiles, gene expressions, and antioxidant enzymes in halophyte *Suaeda salsa*. J Plant Growth Regul.

[36] Liu XL, Yang CY, Zhang LB, Li LZ, Liu SJ, Yu JB, Liu LP, Zhou D, Xia CH, Zhao JM, Wu HF (2011). Metabolic profiling of cadmium-induced effects in one pioneer intertidal halophyte *Suaeda salsa* by NMR-based metabolomics. Ecotoxicology..

[37] Wu H, Liu X, Zhao J, Yu J, Pang Q, Feng J (2012). Toxicological effects of environmentally relevant lead and zinc in halophyte *Suaeda salsa* by NMR-based metabolomics. Ecotoxicology..

[38] Liu X, Wu H, Ji C, Wei L, Zhao J, Yu J (2013). An integrated proteomic and metabolomic study on the chronic effects of mercury in *Suaeda salsa* under an environmentally relevant salinity. PLoS One.

[39] Chen W, Gong L, Guo Z, Wang WS, Zhang HY, Liu XQ, Yu SB, Xiong LH, Luo J (2013). A novel integrated method for large-scale detection, identification, and quantification of widely targeted metabolites: application in the study of rice metabolomics. Mol Plant.

[40] Bylesjö M, Rantalainen M, Cloarec O, Nicholson JK, Holmes E, Trygg J (2006). OPLS discriminant analysis: combining the strengths of PLS-DA and SIMCA classification. J Chemom.

[41] Flowers TJ, Colmer TD (2008). Salinity tolerance in halophytes. New Phytol.

[42] Li X, Liu Y, Chen M, Song YP, Song J, Wang BS, Feng G (2012). Relationships between ion and chlorophyll accumulation in seeds and adaptation to saline environments in *Suaeda salsa* populations. Plant Biosyst.

[43] Munns R, Tester M (2008). Mechanisms of salinity tolerance. Annu Rev Plant Biol.

[44] Song J, Zhou JC, Zhao WW, Xu H, Wang FX, Xu YG, Tian CY (2016). Effects of salinity and nitrate on production and germination of dimorphic seeds applied both through the mother plant and exogenously during germination in *Suaeda salsa*. Plant Spec Biol.

[45] Song J, Feng G, Tian CY, Zhang FS (2006). Osmotic adjustment traits of *Suaeda physophora Haloxylon ammodendron* and *Haloxylon persicum* in field or controlled conditions. Plant Sci.

[46] Cao R, Liang Z, Wu Y, Zhang L, Kang S (2004). The change of amino acids in leaves of maize seedlings under alternative split--root osmotic stress. Agric Res Arid Areas (in Chinese with English abstract).

[47] Moura AP, Ribeiro CAJ, Zanatta Â, Busanello ENB, Tonin AM, Wajner M (2012). 3-Methylcrotonylglycine disrupts mitochondrial energy homeostasis and inhibits synaptic Na^+^, K^+^-ATPase activity in brain of young rats. Cell Mol Neurobiol.

[48] Hou Q, Ufer G, Bartels D (2016). Lipid signalling in plant responses to abiotic stress. Plant Cell Environ.

[49] Sui N, Li M, Li K, Song J, Wang BS (2010). Increase in unsaturated fatty acids in membrane lipids of *Suaeda salsa* L. enhances protection of photosystem II under high salinity. Photosynthetica..

[50] Sui N, Han GL (2014). Salt-induced photoinhibition of PSII is alleviated in halophyte *Thellungiella halophila* by increases of unsaturated fatty acids in membrane lipids. Acta Physiol Plant.

[51] Sui N, Tian SS, Wang WQ, Wang MJ, Fan H (2017). Overexpression of glycerol-3-phosphate acyltransferase from *Suaeda salsa* improves salt tolerance in Arabidopsis. Front Plant Sci.

[52] Zhao YQ, Yang Y, Song YP, Li Q, Song J (2018). Analysis of storage compounds and inorganic ions in dimorphic seeds of euhalophyte *Suaeda salsa*. Plant Physiol Bioch.

[53] Blanchard H, Pédrono F, Boulier-Monthéan N, Catheline D, Rioux V, Legrand P (2013). Comparative effects of well-balanced diets enriched in α-linolenic or linoleic acids on LC-PUFA metabolism in rat tissues. Prostag Leukotr Ess.

[54] Hu FB, Stampfer MJ, Manson JE, Rimm EB, Wolk A, Colditz GA, Hennekens CH, Willett WC (1999). Dietary intake of α-linolenic acid and risk of fatal ischemic heart disease among women. Am J Clin Nutr.

[55] Sinclair AJ, Attar-Bashi NM, Li D (2002). What is the role of α-linolenic acid for mammals?. Lipids..

[56] Jiang XL, Wei D (2010). Analysis of lipid in *Chlamydomonas nivalis* to salt stress by GC/MS. Sci Technol Food Ind (in Chinese with English abstract).

[57] Li H, Wang X, Li Y, Li P, Wang H (2009). Polyphenolic compounds and antioxidant properties of selected China wines. Food Chem.

[58] Robbins RJ (2003). Phenolic acids in foods: an overview of analytical methodology. J Agr Food Chem.

[59] Wang A, Li R, Ren L, Gao XL, Zhang YG, Ma ZM, Ma DF, Luo YH (2018). A comparative metabolomics study of flavonoids in sweet potato with different flesh colors (*Ipomoea batatas* (L.) lam). Food Chem.

[60] Ding HR, Hong L, Yang ZQ, Wang M, Wang K, Zhu XM (2008). Progress of study on halophyte *Suaeda salsa*. Acta Agric Jiangxi (in Chinese with English abstract).

[61] Oueslati S, Trabelsi N, Boulaaba M, Legault J, Abdelly C, Ksouri R (2012). Evaluation of antioxidant activities of the edible and medicinal *Suaeda* species and related phenolic compounds. Ind Crop Prod.

[62] Maisuthisakul P, Suttajit M, Pongsawatmanit R (2007). Assessment of phenolic content and free radical-scavenging capacity of some Thai indigenous plants. Food Chem.

[63] Murakami A, Ashida H, Terao J (2008). Multitargeted cancer prevention by quercetin. Cancer Lett.

[64] Qiu Y, Liu Q, Beta T (2010). Antioxidant properties of commercial wild rice and analysis of soluble and insoluble phenolic acids. Food Chem.

[65] Rice-Evans CA, Miller NJ, Paganga G (1996). Structure-antioxidant activity relationships of flavonoids and phenolic acids. Free Radical Bio Med.

[66] Sroka Z, Cisowski W (2003). Hydrogen peroxide scavenging antioxidant and anti-radical activity of some phenolic acids. Food Chem Toxicol.

[67] Wang B, Lüttge U, Ratajczak R (2004). Specific regulation of SOD isoforms by NaCl and osmotic stress in leaves of the C3 halophyte *Suaeda salsa* L. J Plant Physiol.

[68] Li K, Pang CH, Ding F, Sui N, Feng ZT, Wang BS (2012). Overexpression of *Suaeda salsa* stroma ascorbate peroxidase in *Arabidopsis* chloroplasts enhances salt tolerance of plants. S Afr J Bot.

[69] Song J, Chen M, Feng G, Jia YH, Wang BS, Zhang FS (2009). Effect of salinity on growth ion accumulation and the roles of ions in osmotic adjustment of two populations of *Suaeda salsa*. Plant Soil.

[70] Liu QQ, Liu RR, Ma YC, Song J (2018). Physiological and molecular evidence for Na^+^ and CI^−^ exclusion in the roots of two *Suaeda salsa* populations. Aquat Bot.

[71] Song J, Shi GW, Gao B, Fan H, Wang BS (2011). Waterlogging and salinity effects on two *Suaeda salsa* populations. Physiol Plantarum.

[72] Shao Q, Han N, Ding T, Zhou F, Wang BS (2014). SsHKT1; 1 is a potassium transporter of the C3 halophyte *Suaeda salsa* that is involved in salt tolerance. Funct Plant Biol.

[73] Han N, Shao Q, Bao H, Wang BS (2011). Cloning and characterization of a Ca^2+^/H^+^ antiporter from halophyte *Suaeda salsa* L. Plant Mol Biol Rep.

[74] Fraga CG, Clowers BH, Moore RJ, Zink EM (2010). Signature-discovery approach for sample matching of a nerve-agent precursor using liquid chromatography-mass spectrometry XCMS and Chemometrics. Anal Chem.

[75] Eriksson L, Andersson PL, Johansson E, Tysklind M (2006). Megavariate analysis of environmental QSAR data. Part I–A basic framework founded on principal component analysis (PCA) partial least squares (PLS) and statistical molecular design (SMD). Mol Divers.

[76] Kanehisa M, Goto S (2000). KEGG: Kyoto encyclopedia of genes and genomes. Nucleic Acids Res.

